# Shape Memory Alloy Helical Microrobots with Transformable Capability towards Vascular Occlusion Treatment

**DOI:** 10.34133/2022/9842752

**Published:** 2022-07-06

**Authors:** Hehua Zhang, Borui Xu, Yi Ouyang, Yunqi Wang, Hong Zhu, Gaoshan Huang, Jizhai Cui, Yongfeng Mei

**Affiliations:** ^1^Department of Materials Science, Fudan University, Shanghai 200438, China; ^2^Shanghai Frontiers Science Research Base of Intelligent Optoelectronics and Perception, Institute of Optoelectronics, Fudan University, Shanghai 200433, China; ^3^Yiwu Research Institute of Fudan University, Yiwu 322000, Zhejiang, China; ^4^International Institute for Intelligent Nanorobots and Nanosystems, Fudan University, Shanghai 200438, China

## Abstract

Practical implementation of minimally invasive biomedical applications has been a long-sought goal for microrobots. In this field, most previous studies only demonstrate microrobots with locomotion ability or performing a single task, unable to be functionalized effectively. Here, we propose a biocompatible shape memory alloy helical microrobot with regulative structure transformation, making it possible to adjust its motion behavior and mechanical properties precisely. Especially, towards vascular occlusion problem, these microrobots reveal a fundamental solution strategy in the mechanical capability using shape memory effect. Such shape-transformable microrobots can not only manipulate thrust and torque by structure to enhance the unclogging efficiency as a microdriller but also utilize the high work energy to apply the expandable helical tail as a self-propulsive stent. The strategy takes advantage of untethered manipulation to operate microsurgery without unnecessary damage. This study opens a route to functionalize microrobots via accurate tuning in structures, motions, and mechanical properties.

## 1. Introduction

Biomedical microrobot has been widely accepted as a promising in vivo technology for minimally invasive procedures [1–4]. Despite the small scale of microrobot limits it to perform versatile tasks independently similar with a traditional robot, microorganism in nature provides diverse strategies to actively swim, sense, and act without a complex nervous system [5–7]. Specifically, microorganism capable of active transformation proffers a methodology to enrich functionalities via reacting to signals in real time [8–10]. Inspired by that, several smart material and structure models have been applied to artificial microrobot [11–13], including temperature-responsive polymers [14], hydrogels [15], shape memory alloy [16], light-responsive liquid crystal elastomers [17], graphene [18], programmable magnetic particles [19, 20], and configurable swarms [21]. However, the transformable ability of microrobot mostly serves as actuation or propulsion mechanism for valid motion, and additional modification is still required to realize specific function.

In the biomedical field, vascular occlusion which is mainly characterized by blood clot and plaques becomes a highly hazardous threat to human health, including heart attack, stroke, renal artery disease, or stenosis. At present, the clinical treatment containing drug therapy, vascular bypass graft, and percutaneous transluminal angioplasty exists risk in unnecessary bleeding and damage [22]. At the meantime, in the classic film Fantastic Voyage, the plot that tiny robot submerges into human blood vessel to break the blood clot seems to be a tantalizing inspiration [23]. As profitable solution for invasive vascular procedure problem, the revolutionary medical technology of practical microrobots has received considerable critical attention [24, 25], mainly pharmaceutical and mechanical techniques towards blood clots [26–28] and novel stent towards narrow vascular [29–31].

Here, we develop a shape memory alloy (SMA) microrobot composed of a NiTi-wire tail and a magnetic head, capable of performing multitasks in optimal manner towards vascular occlusion treatment. Under rotation magnetic field, the helical microrobot is actuated to propel and steer in viscous fluid. The helical tail made of SMA endows the microrobot with manageable structure transformation triggered by heating, leading to the desired translational locomotion shifting due to the highly correlation between the motion behavior and helical structure. Based on the transformable helical microrobot model, we propose targeted strategies for vascular occlusion diseases by in vitro experiments. For blood clot problem, the microrobot is investigated to enhance mechanical unclogging effect with shape transformation during the procedure. Besides, the self-propelled microrobot is implemented for plaques problem via triggered expansion by transient heat stimulus once it is guided to the target area. This work presents a practical microrobot functionalized by its reliable transformable capability in a wide range, advancing microrobots of carrying out versatile and sophisticated tasks.

## 2. Results

### 2.1. Fabrication and Transformable Locomotion

**Figure 1:**
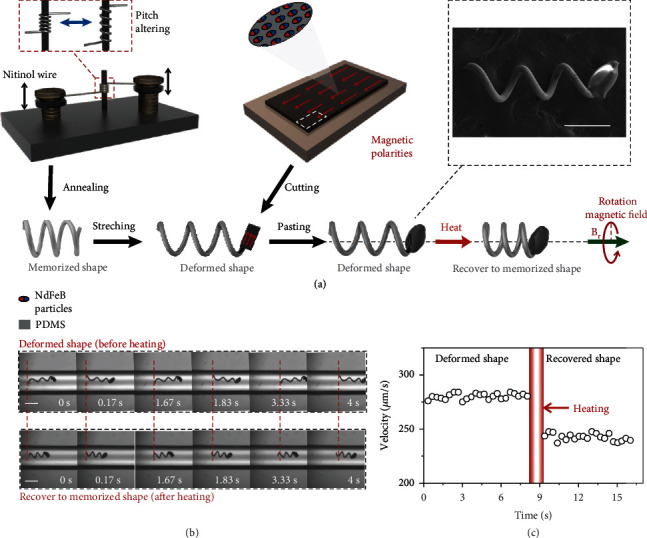
Concept of magnetic helical microrobot with transformable capability. (a) Schematic diagram of the fabrication strategy and transformable motion principles of helical microrobot. The detail of assembling process is described in materials and methods. The assembled microrobot consists of NdFeB particles/PDMS head and NiTi helical tail. The shape-memory helical microrobot is propelled by an external rotation magnetic field. Controllable body shape transformation based on shape memory effect triggered by heating leads to the transformation of helical microrobot locomotion. The top left inset depicts that pitch is altered by adjusting the position of the screws. The top right inset depicts the SEM image of a microrobot. Scale bar is 300 *μ*m. (b) Image sequences showing the moving performance before and after the shape transformation. Scale bar is 500 *μ*m. (c) Velocity of microrobot overtime in response to shape transformation.

The microrobot consists of a polydimethylsiloxane (PDMS) head with magnetic particles and SMA tail in helical geometry. Here, the helical body is conducted by a NiTi wire (top left panel of Figure 1(a)). The NiTi wire was wound around the central tungsten axis and adjusted by changing the axial relative position of the two screws to desired pitch. Then, the fixed NiTi helical structure was annealed to obtain the memorized shape of SMA helical tail. And it was further turned into the deformed shape by external force. Meanwhile, the magnetic head was cut from a PDMS film embedded with aligned NdFeB particles (top middle panel of Figure 1(a)). The hysteresis loop of obtained magnetic head substantiates the response capability to external magnetic field (Supporting Information, Figure S1). Subsequently, the magnetic head was stuck to the helical tail with glue. As the orientation of the head is redirected along the long direction of the helical hail and the magnetization of the parallel to the adjacent body wire and misaligned with the helix axis and magnetic field. Besides, the head instinctively performed to an ellipsoidal shape due to the surface tension of the glue during solidification. The inset of Figure 1(a) presents successful fabrication of SMA helical microrobot.

Under a rotating magnetic field generated by Helmholtz coils (Supporting Information, Figure S2), the helical microrobot is propelled forward in a rotating manner. The magnetic head responding to the rotating magnetic field offers magnetic torque for propulsion, and the helical tail converses the rotational motion to translational propulsion [32, 33]. Then, the swimming helical microrobot in deformed shape is triggered to recover to memorized shape by heating. In low Reynolds number environment, the manageable structure transformation of SMA helical microrobot leads to the transformation of translational locomotion (Figure 1(b) and Movie S1). In a polyvinyl acetate (PVA) solution with viscosity of 4.7 MPa·s similar to blood, the SMA helical microrobot is propelled inside a capillary. After heating, the helical tail transforms to shorter in 0.2 s, and the velocity decreases about 30 *μ*m/s simultaneously (Figure 1(c)). Such speed variation reflects the valid altering in microrobot's motion.

### 2.2. Shape Memory Characteristics of the Helical Tail

**Figure 2:**
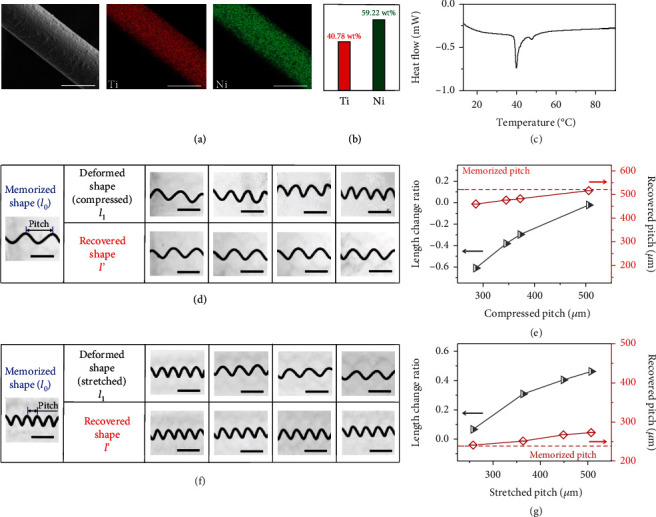
Characterization of the shape-memory helical body of microrobot. (a) SEM image and EDS result of annealed NiTi wire. Scale bar is 50 *μ*m. (b) Weight ratio of Ni and Ti element. (c) DSC result of annealed NiTi wire. (d) Shape memory performance of NiTi helical body when recovered from compressed states. Scale bar is 500 *μ*m. (e) Recovery strain and pitch as a function of different compressed pitches. (f) Shape memory performance of NiTi helical body when recovered from different stretched state. Scale bar is 500 *μ*m. (g) Recovery strain and pitch as a function of different stretched pitches.

Helical tail with shape memory effect governs the precise transformation capability of SMA helical microrobot. As illustrated in the fabrication process, the NiTi wire is constrained into a helical geometry and then annealed to memorize its shape. The SEM image of annealed SMA helix is shown in Figure 2(a), together with the energy dispersive spectrometer (EDS) results of Ni and Ti element distribution. The element weight ratio of Ni is 59.22% and Ti is 40.78% after annealing (Figure 2(b)), of which the Ti component is slightly reduced compared with the NiTi wire before annealing (Supporting Information, Figure S3A). Furthermore, the phase transition temperature of annealed NiTi wire was characterized by differential scanning calorimetry (DSC), revealing that, during heating process, annealed NiTi wire completes phase transition in 45.56°C, and the austenite peak is obtained as Ap = 39.85°C (Figure 2(c)). As a comparison, the phase transition temperature of unannealed NiTi wire is measured as Ap = 48.52°C (Figure S3B). The decrease of transition temperature after annealing attributes to the evaporation of Ti element during heat treatment.

To explore the shape memory characteristics of annealed NiTi helix, a set of deformation tests followed by heating to recover shape were performed. Firstly, the helix with the pitch of 522.1 *μ*m in memorized shape was compressed to different lengths (deformed shape in Figure 2(d)). The deformed helix was then placed on a hot plate with temperature at 65°C to trigger the phase transition (recovered shape). A clear difference between deformed and recovered shapes indicates that shape memory effect induces shape transformation in a wide range (Figure 2(d)). Figure 2(e) gives the measured pitch variation in these circumstances. Here, the length change ratio (LCR) is the length variation between recovered and deformed shape, defined as

(1) $$\begin{array}{c} \text{LCR}=\frac{\left(l^{\prime }-l_{1}\right)}{l_{1}}, \end{array}$$

where *l*_1_ is the deformed pitch length and *l*′ is the recovered pitch length. All compressed SMA helices with LCR up to 60% can recover to their memorized shape. However, small amount of irrecoverable deformation remains. It can be deduced that for a compressed deformation, the annealed NiTi helix exhibits good shape recovery performance as the irrecoverable deformation ratio is less than 10%. Similar situation is also observed in the SMA helix under stretched deformation (Figures 2(f) and 2(g)). According to these results, the controllable shape transformation of the SMA helical tail with helix angle ranging from 49.2° to 71.7° is robust based on shape memory effect. It is worth noting that the temperature of transformation for NiTi wire is about 45.56°C in this work, and the triggering method is particularly important for target biomedical application. For the problem, we consider the localized hyperthermia technology which has proven to be a well-established cancer treatment by destroying the cancer cells. Until now, many techniques have realized heat-delivery locally involving focused ultrasound, near infrared, magnetic hyperthermia, and infusion of warmed liquids. We believe local heating of local tissue to 45.56°C could be achieved with the aid of these technologies.

### 2.3. Regulative Propulsion Behavior under Rotational Magnetic Field

**Figure 3:**
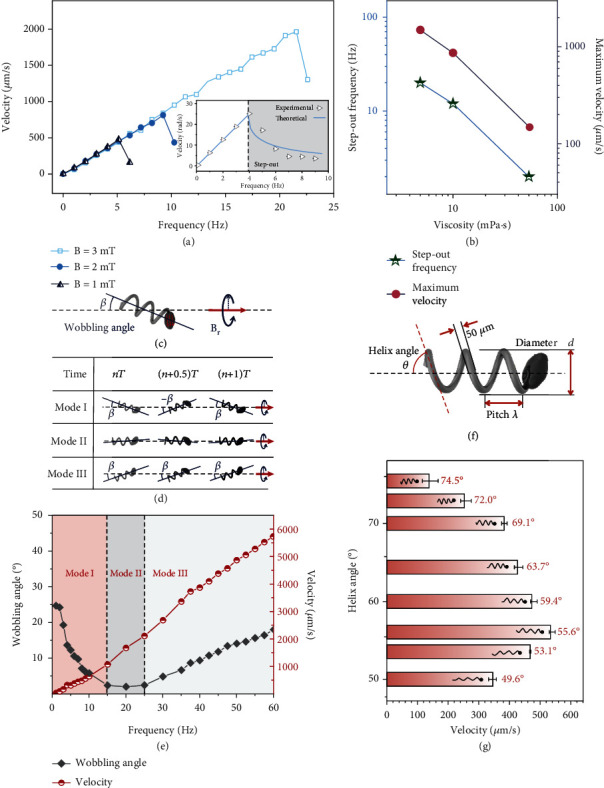
Locomotion behavior of SMA helical microrobot. (a) Velocity with response to different rotation frequency. The inset shows that the experimental and theoretical results of angular velocity related to external field frequency both in synchronous and step-out region. (b) Dependence of step-out frequency and maximum velocity on the viscosity of liquid viscosity. (c) Schematic illustrating the wobbling locomotion of helical microrobot. *β* is the angle between the axis of wobbling gaits and the axis of propulsion locomotion. (d) Optical images showing three gaits of magnetic helical microrobot. *T* is a period of rotation. (e) Variation of wobbling angle and velocity as a function of frequency. The region in different colors refers to different gaits. The density of magnetic field is 10 mT. (f) Schematic presenting structure parameters of a shape-memory helical microrobot. (g) Effect of the helix angle on the velocity when driven by 6 Hz magnetic field with density of 6 mT.

The propulsion behavior of SMA helical microrobot is controlled by external rotating magnetic field properties.  Figure 3(a) shows the velocity with response to the rotation frequency of magnetic field when applying magnetic field intensity is 1 mT, 2 mT, and 3 mT. The velocity is proportional to the rotation frequency on condition of lowering a certain rotation frequency named as step out frequency. This is similar to the pattern found in the work of other helical microrobot [34]. And the velocity decreases drastically once beyond step out frequency in which microrobot loses its synch with the magnetic field due to insufficient torque (inset of Figure 3(a)). Based on the theory of rotating permanent magnets [32], the calculated angular velocity is in good agreement with experimental results. In addition, higher magnetic field intensity directly increases the step out frequency due to the stronger torque supply, yet conducts no effect on the velocity before the step out condition.

Figure 3(b) presents the step-out frequency and maximum velocity of a SMA helical microrobot in liquid environment with different viscosity at 4.5, 10, and 50 MPa·s, which are brought from Supporting Information, Figure S4. It is clearly shown that both step-out frequency and maximum velocity decrease with the rising viscosity. Specifically, it is noticed that the step-out frequency and maximum velocity keep linear relationship with fluid viscosity, reflecting that both are inversely proportional to the fluid viscosity. This relationship is attributed to the variation in fluidic drag torque where viscosity works as a multiplying coefficient [35]. The decrease of step out frequency is because more viscous liquid environment requires more magnetic torque to supply synchronous spiral motion. Besides, considering the proportional relation between the velocity and rotation frequency, larger step-out frequency refers to the higher velocity the microrobot can reach.

Additionally, an attractive phenomenon is observed that the SMA helical microrobot moves in frequency-dependent modes before step out condition. To clarify these different modes, a model containing wobbling angle is demonstrated in Figure 3(c). Here, the wobbling angle *β* is defined as the angle between the helical axis and the translational motion direction. There exist three modes of motion determined by rotating magnetic field frequency (Figure 3(d) and Movie S2). At mode I, the axis of the helical microrobot keeps spinning for 360° in every rotation period, around translational motion direction at a wobbling angle. At mode II, the axis of helix tail keeps constant and coincident with translational motion direction. As for the mode III, the helical microrobot starts to keep a wobbling angle with translational motion direction but helix tail axis still performs no spinning.

Detailed investigation on the relation between observed modes and rotating magnetic field frequency is shown in Figure 3(e). The applied magnetic field intensity is 15 mT, which is sufficient for microrobot to keep sync with rotating magnetic field within the frequency range up to 60 Hz. It can be concluded that the helical microrobot stays in mode I as the frequency is lower than 15 Hz. And the wobbling angle decreases with increasing frequency. As the frequency increases to 15 Hz, the wobbling angle gets close to zero, leading to a stable movement as mode II. When the frequency is higher than 25 Hz, the helical microrobot moves in mode III, and the wobbling angle slightly increases with the rising frequency. Interestingly, while the helical microrobot performs different modes in different ranges of frequency, the velocity is proportional to the rotation magnetic field frequency. In a word, with increase of frequency, demand for torque is rising in response, that is, even before step-out circumstance (magnetic torque is sufficient for sync rotation motion), the external torque still impacts the locomotion modes by the difference between supply and demand of torque. These different modes of helical microrobot motion presents a detailed alteration of navigation strategies, which is expected to be applied in different tasks.

Translational motion of SMA helical microrobot is converted by the rotation of the attached rigid chiral tail. A simple relation between its main structure parameters including pitch (*λ*), diameter (*d*), and helix angle (*θ*) (Figure 3(f)). Helix angle is written as

(2) $$\begin{array}{c} \tan\;\theta =\frac{\pi d}{\lambda }. \end{array}$$

The velocity of SMA microrobot varies when the helix angle ranges from 49.6° to 74.5°. The frequency and intensity of rotation magnetic field are set as 6 Hz and 6 mT, respectively (Figure 3(g)). The tendency that the velocity increases first and then deceases is attributed to the relationship between geometry-dependent viscous resistance and magnetic torque. Due to the contribution of helix structure on propulsion locomotion, the variation of helical tail structure based on shape memory effect is expected to impact on the motion behavior enormously. The heating process is conducted by a hot water drop, which is enough to guarantee the full phase transition of SMA helical microrobot inside the capillary (Supporting Information, Figure S5). With different fabrication process, the SMA helical microrobots transform into a longer or shorter shape and thus alter the moving velocity as well (Supporting Information, Figure S6 and Movie S3). In detail, the shifting of the helix angle was designed, respectively, to distribute in the increasing phase, decreasing phase, or across the peak of the curve in Figure 3(g), thus realizing the arbitrary adjustment of propulsion velocity. Besides, the propulsion modes can also be tuned by helix structure transformation and misalignment angle shifting (Supporting Information, Figure S7 and Movie S4).

### 2.4. Microdriller-Enhanced Mechanical Unclogging Ability via Shape Transition

**Figure 4:**
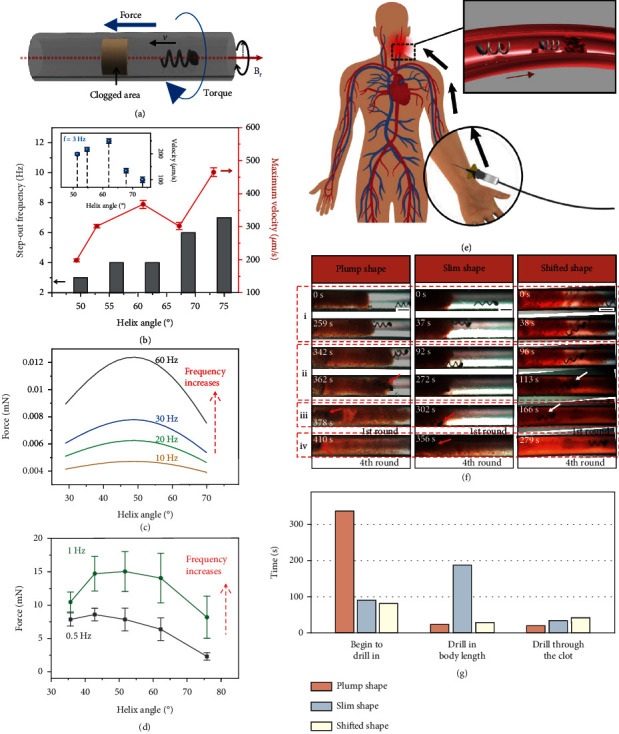
Enhanced mechanical unclogging effect of microdriller by shape transformation. (a) Schematic indicating that the force and torque generated by the rotation of microrobot. (b) Step-out frequency and corresponding maximum velocity with respect to helix angles under 5 mT magnetic field. The inset shows the velocity of microrobot with different helix angle where the frequency of magnetic field is 3 Hz. (c) Calculation result of the force as a function of helix angle. (d) Experimental results of the force with different helix angle of a scaled-up NiTi helix. The wire diameter is 0.3 mm, and the helix diameter is 5 mm. (e) Schematic shows the process of microdriller implement and operation. The microrobot is injected into artery and driven to lesions under rotation magnetic field. The microdriller optimizes the unclogging effect via altering its structure by local heating. (f) Image sequence indicating unclogging performance of helical microrobots in a plump shape, slim shape, and a microrobot transforming from slim to plump shape. The unclogging process is divided into four stages: (i) reaching the location and beginning to drill in, (ii) DRILLING into the clot with one body length depth, (iii) drilling through the clot, and (iv) repeating drilling for four times. Heating process for shape transformation is conducted at the beginning of stage ii. Scale bar is 500 *μ*m. (g) Statistics of time cost in different stages of three unclogging microrobots.

In low Reynolds number environment, a rigid rotating flagellum generates force and torque according to the resistive force theory which is developed to describe swimming driven by a rotating helical flagellum. The induced mechanical effect by swimming motion facilitates the local operation of microrobot in narrow and confined area without tethered manipulation. Here, we demonstrate a potential application as a helical microdriller and enhances its mechanical unclogging effect to penetrate clogged area in blood vessel (Figure 4(a)). To determine the relationship between microrobot structure and mechanical effects, especially the force and torque derived by swimming motion, experiments and calculation have been developed and introduced.

As a promising expectation, these microdrillers can be injected by syringe into the artery, driven to lesions under the guidance of external rotation magnetic field [36], and designed to perform unclogging task (Figure 4(e)). We initially tried to figure out the solution of the helical microrobot when faced with clogged area. We set three relative slight blockings (gelatin) from small to large in the capillary tube and the helical microrobot was driven under constant magnetic field (7 Hz, 5 mT). As shown in Movie S5 and Figure S9, with the mechanical affections, helical microrobot is capable of swimming inside the vessel containing various obstacles as clustered blood cells. The microrobot exhibits three different ways related to the size of blockings due to the difference in the relative magnitude of friction and adhesion, which includes pushing away, passing by, and drilling across. It can be concluded that the helical microrobot is able to fix the potential obstacle problem in a certain degree without optimized, which is also displayed by Liu et al. [37]. However, it takes a minute to drill across the third blocking which is actually a small clogging area. Thus, to improve the efficiency of unclogging process by helical microrobot, we plan to optimize in different aspects.

Firstly, we consider to optimize the torque. The magnetic torque generated by magnetic field is supplied to maintain the rotation of the microrobot for translational movement, and the microrobot would lose synchronization with the rotating magnetic field when the torque is not sufficient to balance viscous force. As discussed above, the step-out frequency decreases sharply when the viscosity increases. In subsequent experiment, we used 9% gelatin to model the blood clot which is jelly-like texture. We could regard the clogged area as an extremely viscous environment, in which continuous motion is almost impossible. Thus, fewer step-out behavior and greater rotation ability is an imperative goal for unclogging. According to our fabrication process, the helix angle of SMA helical microrobot is altered during the shape transformation, while the number of spirals remains constant. Therefore, the effect of helix angle on the step-out frequency and corresponding maximum velocity is studied with the magnetic field intensity of 5 mT (Figure 4(b)). The microrobot with bigger helix angle results in higher step-out frequency. Considering that the bonded head is always parallel to the body wire, bigger helix angle refers to larger misalignment angle. For our SMA helical microrobot, the magnetic head determines the supply of magnetic torque according to the equation written as *τ* = *m* × *B*, where *τ* is the torque, *B* is the external magnetic field intensity, and *m* is the magnetic moment of magnetic head. Therefore, the magnetic torque supply increases with rising helix angle as the projection of magnetic moment on rotation plane gets larger, leading to a higher step-out frequency. However, the microrobot with higher step-out frequency not always processes higher maximum velocity. The reason was explained in the inset of Figure 4(b), in which the velocity of the shape memory helical microrobots with different five helix angles is presented when applying 3 Hz rotation magnetic field. Microrobot with helix angle of 62° propels fastest, so it reaches higher maximum velocity with a low step-out frequency.

Secondly, puncture force is also taken into consideration. The high-speed rotating spiral microrobot performs similar with a microdriller, and the puncture force is a significant mechanical parameter. Johnson slender body theory is utilized to expose the impact of structure on the force (Figure 4(c)). The thrust force increases first and then decreases with rising helix angle and reaches a maximum at approximately 48°. The force also increases as the microrobot rotates at a higher frequency. Besides, similar tendency can be observed from experiment focusing on a scaled-up microhelix (Figure 4(d); parameters can be found in Methods), which is shown in Figure 4(d). Given the above, the microhelix provides more puncture force when the helix angle reaches about 50° and microhelix with bigger helix angle is less likely to step out. Based on that, we can enhance the unclogging effect by shape shift real-time.

As studied, microrobot with helix angle of about 53° exerts the biggest thrust, and the larger the helix angle, the smaller the magnetic torque required for motion. Accordingly, we designed the transformable microrobot with helix angle shifting from 53° to 69° and set comparison test with other two microrobot which have constant structure. In order to demonstrate the mechanical unclogging effect, gelatin artificial clot was applied to mimic clogging blockage in blood vessel (Supporting Information, Figure S10). Given that the rigid tail obtains stronger pressure compared to rounded head, all the unclogging experiment were conducted by swimming towards the direction of tail. Microrobots with constant helix angle of 69° (plump shape) and 53° (slim shape) and SMA helical microrobot (helix angle transforms from 53° to 69°) were guided to pass through the artificial clot where all applying rotating magnetic fields set as 20 Hz and 20 mT (Figure 4(f) and Movie S6). The plump microrobot finishes the whole task in 410 s while the slim one completes in 365 s. In contrast, in the same task implemented by the SMA helical microrobot, transforming from plump to slim shape as soon as drilling into the clogging blockage, the highly effective unclogging process ends in 245 s.

To clearly compare different helical microrobots, we divided the whole unclogging process into four stages as (i) attaching the clot to drill, (ii) drilling in with one body length depth, (iii) drilling through the clot, and (iv) breaking the clot (Supporting Information, Figure S11). To analyze the unclogging performance of each helical microrobot, the time cost in every stage is listed in Figure 4(g). It is noticed that the helical microrobot in slim shape gets faster to thrust into the blockage the start the drilling process, while the plump one performs better in the drilling process. This difference is caused by the different determinants in different stages. The magnitude of puncture force is a critical factor to start the drilling process, while the magnetic torque determines whether the microrobot can keep rotating and meanwhile breaking clots during drilling. By optimizing the thrust and torque magnitude through shape transformation, the SMA helical microrobot realizes the mechanical unclogging function with enhanced performance.

### 2.5. Self-Propelling Stent towards Plaque Problem

Shape memory alloy, owning advantages of good corrosion resistance and biocompatibility, has been widely implemented in invasive stent implantation surgery to open up clogged arteries [38]. However, the intervention operation might lead to damage in the catheter-placement process, which hugely depends on the operational proficiency of physician. Untethered helical microrobot is regarded as a noninvasive method because of the flow layer generated during propulsion that keeps microrobots from the contact with vessel wall (Supporting Information, Figure S12).

**Figure 5:**
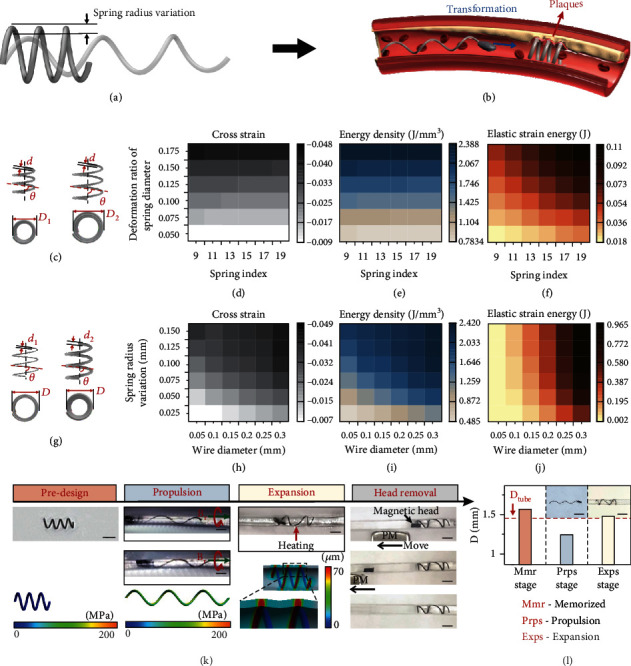
Thermal responsive self-propelling stent towards plaque problem. (a) Schematic showing the helix radius variation occurred during SMA tail deformation. (b) Schematic exhibiting the strategies of shape memory helical microrobot towards self-propelled stent. (c) Simulation model of helices with same wire diameter *d* and helix angle *θ*. (d–f) Impact of spring index and deformation ratio of diameter in the model of (c) on crossstrain, energy density, and elastic strain energy, respectively. (g) Simulation model of helices with same helical outer diameter and helix angle. (h–j) Impact of wire diameter and helical radius variation in the model of (g) on crossstrain, energy density, and elastic strain energy, respectively. (k) Image sequence of the realization of a self-propelling stent. The procedure includes predesign, propulsion, expansion, and removal of magnetic head stages. The bottom left panels are the simulation result of the predesign and expansion process. The color mapping in first two panels refers to the stress, and one in the last panel refers to radial displacement. Scale bar is 2 mm. (l) Photograph and statistics recording the diameter change of stent before and after expanding. Scale bar is 4 mm.

Apart from that, a significant variation in diameter occurs within manageable deformation range of the SMA helices (Figure 5(a)), based on which we developed a self-propelling stent towards plaque problem. The SMA helical microrobot propels at small helix diameter and transforms to large diameter responding to real-time heating, converting thermal energy into mechanical energy. Ultimately, the high energy density characteristic enables microrobot to expand and open up the plaques in blood vessel wall (Figure 5(b)). The self-propelling stent can be manufactured in various sizes to be placed in the vasculature and airways. Moreover, they can be coated with diverse drug particles to enable the local delivery of therapeutics through circumferential injections.

The optimized supporting performance of SMA self-propelling stent was investigated by simulation. Two sets of controlled simulation trials were set up to disclose the crosssectional strain, energy density, and stored elastic strain energy as a function of structure parameters during the deformation of SMA stent. Figure 5(c) shows the schematic of first helices controlled trial made from NiTi wire with same wire diameter *d* of 0.1 mm. These helices are manufactured with various diameters yet same helix angle of 70° and deforming with same ratio of helix diameter change in martensitic phase. Spring index (*C*) is written as *C* = *D*/*d*, where *D* is the medium diameter of helix. Deformation ratio of helix diameter (DCR) is written as

(3) $$\begin{array}{c} \text{DCR}=\frac{D_{\text{O}}-D_{\text{o}}^{\prime }}{D_{\text{O}}}, \end{array}$$

where *D*_O_ is the outer diameter of initial helix and *D*_o_′ is outer diameter of deformed helix. Figures 5(d)–5(f) are the effect of spring index and deformation ratio of helix diameter on crossstrain, energy density, and stored elastic strain energy. The results suggest that the crossstrain and energy increase almost linearly with the rate of DCR and are hardly affected by spring index, while the stored elastic strain energy is significantly affected by both. Figure 5(g) is the second controlled trial, including helices with same *D*_O_ and helix angle made from different wires. Helix radius variation (∆*D*_O_) is written as ∆*D*_O_ = *D*_O_ − *D*_o_′. Evolution of crossstrain, energy density, and stored elastic strain energy related to helix radius variation and filament diameter is summarized in Figures 5(h)–5(j). It can be concluded that both of filament diameter and helix radius impact crossstrain and energy density. Besides, for a specific goal diameter to reach, filament diameter provides a greater influence on elastic strain energy.

The realization of a microrobot-based stent includes structure predesign, propulsion to the target area, self-expansion to dilate the plastic capillary, and removal of magnetic head (Figure 5(k) and Movie S7). Here, the SMA helical microrobot is designed with the shape transformable from a long helix to short one to obtain an increasement in helix diameter. As the SMA helical microrobot is guided to the target area of the vessel, transient heating triggers the shape transformation. The shrinkage of the SMA helical microrobot leads to the increase in radius, so the plastic capillary is expanded. Besides, the toxic magnetic head is removed by additional magnetic field while the expanded helix tail left in the plastic capillary as a stent due to the confinement of the capillary wall. Further simulation reflects that stress is stored in the stretched helix tail, which is sufficient to expand the outer capillary (bottom left panels in Figure 5(k)). Figure 5(l) is the photograph and statistics recording the diameter evolution of the self-propulsive stent. The stent is manufactured with outer diameter slightly higher than inner diameter of the plastic capillary. It is then stretched to longer in length and smaller in diameter, sufficient to swim in the capillary. When the stent is inserted and propel to requisite position, phase transition is triggered and stent expands the vessel. The diameter in expansion stage exceeds the diameter of the capillary (1.5 mm), reflecting that an effective expansion of capillary wall is obtained by the self-propelled stent. Moreover, we tested the cytotoxicity of shape memory helical microrobot in condition of incubating at 37°C and 46°C individually to test its biocompatibility both in normal state and transition state. The test results show that the shape memory microrobot has no potential cytotoxicity in both state (Supporting Information, Figure S14).

## 3. Discussion

In summary, we report a biocompatible functionalized shape memory alloy helical microrobot with wide-range active transformation capability providing solution strategies for vascular occlusion problem. The propulsion mechanism based on rotating magnetic field possesses many excellences for biomedical application, including harmless manipulation, low-cost signal generation, and stable motion. The shape memory alloy tail provides shape transformation capacity with helix angle ranging from 49.2° to 71.7°, as well as superiorities of great power density and low trigger temperature, maintaining deformation without constant stimuli. We investigated the translational velocity as respect to external magnetic field, surrounding environment and microrobot structure to draw the locomotion manipulation rules triggered by structure transformation. Furthermore, as a key point for practical operation, the step-out occurrence was discussed from the perspective of magnetic torque supply and demand. As for the external rotating magnetic field, larger magnetic intensity generates more magnetic torque while faster frequency requires more magnetic torque to make the microrobot keep up with the rotation. Besides, when viewed by the structure, our SMA helical microrobot with larger helix angle supplies more magnetic toque due to the increasing angle between magnetization direction and propulsion direction. Based on the investigated content, we demonstrate potential application of the SMA helical microrobot towards vascular occlusion diseases. The hardening of plaque (also called atherosclerosis) in blood vessels, which is mainly composed of fat, cholesterol, and other hydrophobic molecules, leads to the narrow of blood vessel. And blood clots are gel-like clumps in blood vessel that do not dissolve naturally might completely block the vessel. Compared to the clinical treatment, containing statin therapy to lower low-density lipoproteins (LDL) concentration and conventional percutaneous intervention methods by introducing a manually operated tethered device, our untethered microrobot provides treatment strategies without unnecessary bleeding and damage. The enhanced unclogging performance for blood clot problem benefits from the mechanical property alteration generated by structure and locomotion transformation, mainly focusing on the thrust and torque distribution. And a prototype of stent with self-propelled ability takes advantages of the shape memory tail transforming from a propelling component to expanding functional device.

In exception to what we have studied above, the complete operation of biomedical microrobot towards in vivo therapy requires other technologies to give real-time position and precisely guide their navigation. We draw a schematic diagram showing medical operation process assisted by microrobot, shown in Figure S15. The doctor could observe the motion and behavior of magnet-driven microrobot on the computer in real time and also make necessary control operations during the whole process. Thus, imaging and tracking technology is indispensable for further implementation of biomedical microrobot application. Until now, several types of imaging techniques have been developed and tested to visualize microrobot in vivo, including fluorescent imaging, optical coherence tomography (OCT), magnetic resonance imaging (MRI), and photoacoustic (PA) imaging [39].

This work is an example of well-functionalized microrobot characteristics by reliable transformation ability towards vascular occlusion treatment, marking a step towards smart microrobots with realistic functions. The microrobot strategy opens a promising pathway for practical artificial microrobot featuring biocompatibility, precise control, and cost-effectiveness to carry out sophisticated tasks in biomedical applications.

## 4. Materials and Methods

Synthesis of magnetic matrix head: prepolymer (monomer) and curing agent (crosslinker) of a PDMS elastomer were combined in a 10 : 1 mass ratio during preparation (Sylgard 184, Dow Corning). The mixtures of NdFeB magnetic powders (diameter: ~25 *μ*m, Jiangxi Jinli Permanent Magnet Technology Co. Ltd.) and PDMS mixture were poured into a Teflon mold to fabricate a bonded permanent magnet. Before curing, the mixture was vacuumized to remove induced bubbles during mixing. The mixtures were cured at 373 K for 1 h and then removed from the mold. A uniform magnetic field of 3 T of a vibrating sample magnetometer (VSM, Quantum Design) was applied to program the magnetization of the magnet. The magnetic head with specific size (100 × 80 × 60 *μ*m with 40% error range) was constructed by controlling the step blade to cut the cured mixture.

Fabrication of the microrobot. As for the magnetic head, the permanent magnet mixture of NdFeB particles and PDMS was cut into cuboid blocks with the assistance of a stepping motor, and the polarity direction was set as the longest axis direction in order to simplify operation. For helical tail, the original NiTi wire (diameter: 50 *μ*m, nominal transition temperature: 45°C, Shanghai Shengtong Metal Technology Company) was fixed as helix with diameter of about 250 *μ*m. Then, it was annealed (450°C for 1 hour) to memorize its structure, followed by the magnetic head bonding along the helical tail. Strong adhesion glue was employed to assemble microrobot head and tail together assisted by a micromanipulator.

Characteristics: the optical images were captured by electronic microscope OLYMPUS BX51 associated with CCD camera. The SEM image and EDS measurement were performed by Nova NanoSem 450 scanning electron microscope. DSC result of NiTi wire was measured and analyzed by DSC Q2000. Heating curve ranges from 10 to 100°C with rate of 5°C/min.

Recording and analysis of microrobot's motion. The magnetic field control platform (Supporting Information, Figure S2) contains three parts: Helmholtz device composed of three pairs of orthogonal coils and power amplifiers to generate rotating magnetic field, optical microscope to expose microscale behaviors, and CCD camera to record motion videos of 30 frames per second. PVA solution with different viscosity and dimethylsilicone oil (Macklin) were injected into the capillary tube using a syringe, and then, the microrobot was placed into the capillary tube by a micromanipulator. Capillary tube containing microrobot for the motion test was placed on the sample stage in the core of the coils. To analyze the motion and structure transformation, the ImageJ with FIJI package which facilitate scientific image analysis was applied.

Artificial clot: the artificial clot was made of gelatin mixed with glass fibers and PMMA microspheres to simulate the tissues in a blood clot. 0.27 g gelatin powder (Shanghai Fengwei Industry Company), 0.138 g Congo red dye, 0.21 g glass fiber, and 0.02 g microbeads were mixed with 3 mL deionized water. The suspended mixture is heated in a water bath for 20 min at 50°C. After dissolving completely, the mixed solution was cooled down to 2°C and kept for two hours to gel sufficiently. In order to set the gelatin clot into capillary for unclogging experiment, the capillary tube was pricked into the gelatin artificial clot to obtain an intact artificial clot segment remaining in the capillary. The clot segment was carefully pushed to specific location by a metal rod, and PVA solution was injected into both sides of the clot segment utilizing syringe injection.

Analysis of thrust force during rotation: the thrust force of a rotating helical microrobot was calculated based on Johnson slender body theory [40]. Details can be found in Supplementary Note. The dynamic viscosity of fluid was 5 MPa·s. We fixed the total length of the helical microrobot in order to find out the influence of helix angle on the thrust force. Here, the original shape of the helical robot was set as a helix with 150 *μ*m pitch and 125 *μ*m radius. And the number of turns and the radius of SMA wire were fixed as 3 and 25 *μ*m, respectively.

Setup of thrust force measurement: in order to facilitate the measurement, we used a scaled-up helix with diameter of 5 mm and wire diameter of 0.3 mm to rotate at frequency of 0.5 Hz and 1 Hz, respectively, in viscous environment with dynamic viscosity of 50 MPa·s, and there was no translational speed during the test. One end of the tested helix is connected to the motor, and the rest of the helix is submerged in dimethylsilicone oil (Macklin) filled in a tube. The motor is fixed on a slider, providing constant rotation rate to the helix. Axial force caused by the rotating flagellum in low Reynolds number environment is tracked by the load cell that is touched with the end of a rotating motor (Figure S8).

Simulation of self-expanded stent: the simulation of self-propelled stent was conducted by Ansys Workbench. Static structural analysis system was constructed to simulate the pre-design and expansion process. We established a shape memory alloy model for helix and elastic model for capillary tube. The shape memory alloy model includes an isotropic elastic property and shape memory effect data, including Young's modulus of 35 GPa, Poisson's ratio of 0.2, hardening parameter of 500 MPa, reference temperature of 38°C, elastic limit of 120 MPa, temperature scaling parameter of 8.3 MPa/°C, maximum transformation strain of 0.07, martensite modulus of 20 GPa, and lode dependency parameter of 0. The tube is conducted with Young's modulus of 4 MPa. In predesign process, a radial pressure loading was applied first to create a perturbation on the helix. Then, an axial force loading was added at one end of the helix to stretch it to the deformed shape. In expansion process, the contact between helix and tube was set as frictionless, and the deformed helix was placed into the tube by adding a displacement confinement. Then, the temperature of the helix was raised to trigger the shape memory effect.

Cell viability test: negative control: high density polyethylene; positive control: zDEC (manufacturer: Tokyo Chemical Co.); blank control: MEM medium with 10% fetal bovine serum. Mouse fibroblasts L929, cell line from the American Strain Collection CCL1 (NCTC clone 929), were used for this test. The microrobot samples were extracted using MEM medium containing 10% fetal bovine serum and incubated for 24 hours at 37°C in a shaker at 60 rpm. The extracts were checked for changes at the end of the extraction and implemented immediately in the experiment. After the L-929 cells had grown into a monolayer, the original medium was aspirated and 100 *μ*L of different concentrations of the test sample (100%, 75%, 50%, and 25%), empty control solution, negative control solution, and positive control solution were added and incubated at 37°C for 24 hours with 5% CO_2_. 6 parallel samples were made. After 24 h incubation, the 96-well plates were removed, and the cells were observed morphologically under microscope. After 50 *μ*L of MTT (1 mg/mL) was added to each well and incubated in a CO_2_ incubator for 2 hours, the supernatant was discarded, and 100 *μ*L of isopropanol was added to each well to dissolve the crystals. Then, the microplates were shaken for 10 min, and the optical density was measured at 570 nm on an enzyme marker.

## Data Availability

All data is available in the main text or the supplementary materials.

## Supplementary Materials

Supplementary 1 Figure S1: characteristics of magnetic head. Figure S2: experimental setup for rotational magnetic field generation. Figure S3: characterization of original NiTi wire. Figure S4: translational velocity responding to applied frequency when operated in liquid with different viscosity. Figure S5: analysis of heating process by adding a drop of water. Figure S6: real-time locomotion tuning due to shape transformation. Figure S7: gait variation due to shape transformation of SMA helical microrobot. Figure S8: experimental setup of various force measurement related to helix angle. Figure S9: locomotion of rigid helical microrobot in the capillary containing obstacles. Figure S10: photograph of an artificial clot. Figure S11: displacement as respect to time of three unclogging strategies. Figure S12: flow field distribution when the microrobot tail rotates in low Reynolds number environment. Figure S13: experimental result of evolution of work energy with response to spring index. Figure S14: viability of L-929 cells with SMA microrobot. Figure S15: schematic diagram of medical surgery operated by microrobot. Figure S16: modeling of microrobot moving against flowing liquid.

Supplementary 2 Movie S1: SMA helical microrobot capable of transformable ability.

Supplementary 3 Movie S2: the three propulsion modes with respect to frequency under same magnetic intensity.

Supplementary 4 Movie S3: different velocity transformation deduced by different structure transformation.

Supplementary 5 Movie S4: propulsion modes shifting caused by structure transformation.

Supplementary 6 Movie S5: the adaptive strategy of helical microrobot when faced with sighter clogging area.

Supplementary 7 Movie S6: demonstration of enhanced unclogging effect of transformable SMA microdrill.

Supplementary 8 Movie S7: demonstration of self-propelling stent.
