# Supplementary material for: Shape Memory Alloy Helical Microrobots with Transformable Capability towards Vascular Occlusion Treatment
Source: Research (Wash D C). 2022 Jul 6;2022:9842752. doi: 10.34133/2022/9842752 (PMC9297727; doi:10.34133/2022/9842752)
Supplement: Supplementary 1 — Figure S1: characteristics of magnetic head. Figure S2: experimental setup for rotational magnetic field generation. Figure S3: characterization of original NiTi wire. Figure S4: translational velocity responding to applied frequency when operated in liquid with different viscosity. Figure S5: analysis of heating process by adding a drop of water. Figure S6: real-time locomotion tuning due to shape transformation. Figure S7: gait variation due to shape transformation of SMA helical microrobot. Figure S8: experimental setup of various force measurement related to helix angle. Figure S9: locomotion of rigid helical microrobot in the capillary containing obstacles. Figure S10: photograph of an artificial clot. Figure S11: displacement as respect to time of three unclogging strategies. Figure S12: flow field distribution when the microrobot tail rotates in low Reynolds number environment. Figure S13: experimental result of evolution of work energy with response to spring index. Figure S14: viability of L-929 cells with SMA microrobot. Figure S15: schematic diagram of medical surgery operated by microrobot. Figure S16: modeling of microrobot moving against flowing liquid. [file 9842752.f1.docx]

Supplementary Materials

**This file includes:**

Supplementary Text

Figs. S1 to S13

**Other Supplementary Materials for this manuscript include the following:**

Movies S1 to S7

**Supplementary Text**

**Material properties of Shape memory alloy**

Shape memory alloy (SMA) is a traditional smart material that can “memory” its original shape. The behavior is attributed to the microscope transition induced by mechanical stress or temperature changes, which are characterized by shape memory effect (SME) and superelasticity, respectively. The two crystal structures of SMA are called austenite, which is the structure at higher temperatures, and martensite, which is the structure at lower temperatures. The transformation from austenite to martensite is robust and with high energy density, which enables SMA of wide applications in clinical medicine and aerospace. In this work, we utilized the SME of NiTi wire to extend functionalities and optimize effects by precisely control of transformation temperature and structure tuning performance.

In the bottom left panels of Figure 5K, we simulated the stress distribution and displacement of a SMA helical microrobot. Here, the material properties of SMA with shape memory effect is based on the model proposed by Lagoudas [41]. In this model, the strain applied on SMA can be divided into four parts as

$$\varepsilon=\varepsilon_{e}+\varepsilon_{T}+\varepsilon_{t}+\varepsilon_{p}$$

Here, *ε_e_*, *ε_T_*, *ε_t_* and *ε_p_* are the elastic, thermal, transformation, and plastic strain component, respectively. And the stress can be expressed as

$$\dot{\sigma}=E_{\xi}\dot{\varepsilon_{e}}+\alpha_{\xi}\dot{T}+\Omega_{\xi}\dot{\xi}+h_{\xi}\dot{\varepsilon_{p}}$$

Here, *T* is the temperature and *ξ* is the martensite volume fraction. *E_ξ_*, *α_ξ_*, and *h_ξ_* are the bulk modulus, thermal expansion coefficient and hardening modulus related to the martensite volume fraction *ξ*. In Lagoudas modulus, *α_ξ_* is considered as a constant both in martensite and austenite phase. And the bulk modulus *E_ξ_* can be written as

$$E_{\xi}=E_{A}+\xi(E_{M}-E_{A})$$

where *E_A_* and *E_M_* are the bulk modulus of austenite and martensite phase.

As for the transition strain *ε_t_* and plastic strain *ε_p_*, they are given as follows:

$$\dot{\varepsilon_{t}}=\Lambda_{t}\dot{\xi}, \dot{\varepsilon_{p}}=\Lambda_{p}\dot{\bar{\epsilon_{p}}}$$

Here, $\bar{\epsilon_{p}}$ is a measure of the history of plastic strain evolution given as

$$\bar{\epsilon_{p}}=\int_{-\infty}^{t} \left| \dot{\varepsilon_{p}} \right|d\tau$$

And other two parameters are given as

$$\Lambda_{t}=\left\{ \begin{matrix} -\frac{3}{2}\frac{\Omega_{\xi}}{E_{\xi}}\frac{\sigma_{t}}{\sigma_{t}^{eff}} & \dot{\xi}>0 \\ -\frac{\Omega_{\xi}}{E_{\xi}}\frac{\varepsilon_{t}}{\varepsilon_{t}^{eff}} & \dot{\xi}<0 \end{matrix} \right.$$

$$\Lambda_{p}=\frac{3}{2}\frac{\sigma_{p}^{eff}'}{\bar{\sigma}_{p}^{eff}}$$

Here, $\sigma_{t}^{eff}$ and $\varepsilon_{t}^{eff}$ are the effective stress and strain in transition. And $\sigma_{p}^{eff}$ is the effective stress for plastic strain.$\bar{\sigma}_{p}^{eff}$ denotes the *Mises equivalent* of effective stress.

**Simulation of heating process.**

To verify the hot water drop heating process capable of triggering transition sufficiently, we derived a model by Heat Transfer in Solids and Fluids component in Comsol Multiphysics (*SI Appendix*, Figure S6). A silica glass ring hollow pipe body with inner diameter of 0.8 mm and outer diameter of 1 mm was conducted, featuring isobaric heat capacity of 703 J/(kg·K), heat capacity of 1.38 W/(m·K) and density of 2203 kg/m^3^. The liquid inside was set as Newtonian liquid and the laminar flow module was introduced, taking into account the gravity factor. The coefficient of dynamic viscosity was set as 4.7 mPa·s. The original temperature of this system was 293.15 K. The outer wall of the tube was applied with forced convective heat flux (333.15 K) lasting for 0.2 s. This condition was set based on the assumption that the water droplet keeps 60°C and stays around the capillary tube for 0.2 s. Then the tube was set in natural air convection of 293.15 K and the heat flux can be calculated as,

$$q=h(T_{ext}-T)$$

In which *q* is the heat flux, *h* is heat transfer coefficient, *T_ext_* is the temperature of external experiment and *T* is temperature of tube.

**Helix model for thrust force analysis.**

The thrust force of a helix (Figure 4C) is calculated based on the Johnson slender body theory [40]. According to the Johnson slender body, the radius of the cross section along the centerline is determined by following equation.

$$r\left( s \right)=2\epsilon\sqrt{s(L-s)},s\in[0,L]$$

where *s* is the arclength, and $\epsilon$ is the slenderness ratio defined as *r*/*L*. Therefore, the average radius in a Johnson slender body theory model is calculated as *r**π/4.

The position along the centerline is given by ***x***(*s*, *φ*), in a helical structure, the position can be expressed as following.

$$\boldsymbol{x}\left( s,\varphi\right)=(s\cos\theta,R\cos\varphi,R\sin\varphi)$$

Here, *θ* is the pitch angle and *R* is the radius of the helix. And *φ* refers to the phase angle, which is defined as 2π*s/(*L*/*N*). *N* is the number of turns and *L*/*N* refers to the pitch of the helix. Therefore, the position ***x*** can be expressed only by *s* or *φ*. Then, the velocity of a point on the helical surface *s_0_* is given by

$$\mathbf{u}\left( \boldsymbol{x}(s_{0}) \right)=\frac{1}{8\pi\mu}\left\{ -\Lambda\left[ \boldsymbol{f} \right]\left( \boldsymbol{x}(s_{0}) \right)-K\left[ \boldsymbol{f} \right]\boldsymbol{x}(s_{0}) \right\}$$

where ***f*** refers to the force applied on the unit point. The local operator Λ is given by

$$\Lambda\left[ \boldsymbol{f} \right]\left( \boldsymbol{x}(s_{0}) \right)=[-\ln\left( \epsilon^{2}e \right)\cdot\left( \mathbf{I}+\hat{\boldsymbol{s}}(s_{0})\hat{\boldsymbol{s}}\left( s_{0} \right) \right)+2\left( \mathbf{I}-\hat{\boldsymbol{s}}(s_{0})\hat{\boldsymbol{s}}\left( s_{0} \right) \right]\boldsymbol{f}(s_{0})$$

and the integral operator K is given by

$$K\left[ \boldsymbol{f} \right]\left( \boldsymbol{x}(s_{0}) \right)=\int_{0}^{L} \left( \frac{\mathbf{I}+\hat{\boldsymbol{\Delta}}\left( s_{0},s \right)\hat{\boldsymbol{\Delta}}\left( s_{0},s \right)}{\left| \boldsymbol{\Delta}\left( s_{0},s \right) \right|}\boldsymbol{f}\left( s^{'} \right)-\frac{\mathbf{I}+\hat{\boldsymbol{s}}\left( s_{0} \right)\hat{\boldsymbol{s}}\left( s_{0} \right)}{\left| s_{0}-s \right|}\boldsymbol{f}(s_{0}) \right)ds$$

Here, $\hat{\boldsymbol{s}}\left( s \right)$ is the tangential unit vector at ***x***(*s*). $\hat{\boldsymbol{\Delta}}\left( s_{0},s \right)=\boldsymbol{x}\left( s_{0} \right)-\boldsymbol{x}(s)$. $\hat{\boldsymbol{s}}\hat{\boldsymbol{s}}$ and $\hat{\boldsymbol{\Delta}}\hat{\boldsymbol{\Delta}}$ refer to dyadic products. To get the relation between the velocity and force via above equations, we applied finite element method and modified the code provided by Bruce Rodenborn [42]. And the translational velocity was set as zero during the calculation of the thrust force.

**Model of movement capability of helical microrobot.**

First of all, the fluid distribution of a cylindrical channel was carried out. According to related theory and simulation results in **Figure S16**, it is clear that the fluid velocity follows a parabolic distribution. Here, we only consider the distribution in xz plane as a simplification. Therefore, the distribution can be expressed by following equation as we set the x coordinate of the channel center as zero.

$$v_{f}\left( x \right)= 2v_{avg}(1-\frac{x^{2}}{R^{2}})$$

Where *v_avg_* is the average flow velocity, and *R* is the radius of the channel. Assuming that the helical microrobot is moving near the channel boundary, we can compute the average fluid velocity affecting the motion of microrobot by

$$v_{avg}^{fm}=\frac{1}{2r}\int_{-R}^{-R+2r} \left( v_{m}-v_{f} \right)dx$$

*r* is the radius of the helix, and *v_m_* is the velocity of the helical microrobot. Hence, (*v_m_* - *v_f_*) refers to the relative velocity distribution. Based on the relative average velocity $v_{avg}^{fm}$, the relation between external force *F_ex_*, torque *T_ex_*, rotating frequency *f*, and relative velocity can be expressed as[43]

$$\left[ \begin{matrix} F_{ex} \\ T_{ex} \end{matrix} \right]=\left[ \begin{matrix} a & b \\ b & c \end{matrix} \right]\left[ \begin{matrix} v_{avg}^{fm} \\ 2\pi f \end{matrix} \right]$$

Where the constant *a*, *b*, and *c* are the translation parameters in propulsion matrix, which are determined by the microrobot geometric parameters and fluid properties. In our circumstance, the rotating magnetic field only provides rotating torque *T_ex_*, and the rotating frequency is same as the frequency of rotating magnetic field before step-out frequency. Therefore, the equation above can be rewritten as

$$a\cdot v_{avg}^{fm}+b\cdot2\pi f=0$$

$$T_{ex}=v\cdot v_{avg}^{fm}+c\cdot2\pi f$$

According to resistive force theory, the translation parameters of a helical structure with *n* turns is written as [35]

$$a=2\pi nr\frac{\xi_{\parallel}\cos^{2}\theta+\xi_{\perp}\sin^{2}\theta}{\sin\theta}$$

$$b=2\pi nr^{2}(\xi_{\parallel}-\xi_{\perp})\cos\theta$$

$$c=2\pi nr^{3}\frac{\xi_{\parallel}\sin^{2}\theta+\xi_{\perp}\cos^{2}\theta}{\sin\theta}$$

Here, *θ* is the helical angle. ξ_∥_and ξ_⊥_ can be calculated by the formula provided by Lighthill. [44]

$$\xi_{\parallel}=\frac{2\pi\mu}{\ln\left( \frac{0.36\pi r}{r_{w}\sin\theta} \right)-0.5}$$

$$\xi_{\perp}=\frac{4\pi\mu}{\ln\left( \frac{0.36\pi r}{r_{w}\sin\theta} \right)}$$

Here, *μ* is the fluid viscosity. *r_w_* is the wire radius. Based on the equations mentioned above, we calculated the microrobot velocity with respect to the rotating frequency and fluid velocity, and the results are given in Re-Figure 3B. Parameters used in the calculation are given in **Table S1**. The top left part refers to high velocity area, which reflects that the helical microrobot performs rapid translational motion with high frequency and low fluid velocity. The dashed line points out the situation that microrobot rotates without translational motion. And the bottom left part with blue color refers to a backward motion in which situation the microrobot’s propulsion cannot overcome the flowing fluid. According to this phase diagram, it is confirmed from modeling that our microrobot can move forward in a flowing fluid channel with velocity up to 5 mm/s as the step out frequency is more than 60 Hz as we shown in the manuscript.

Furthermore, we give a computational fluid dynamics (CFD) simulation to observe the fluid distribution of a rotating helical microrobot with forward translational motion in a flowing fluid channel. We build a 3D model in COMSOL Multiphysics with rotating machinery interfaces. The average velocity of fluid is 1 mm/s. The rotating frequency and translational speed of microrobot is 20 Hz and 1 mm/s, respectively. It is shown that the rotating helical microrobot moves forward together with surrounding fluid, reflecting its effective motion against flowing fluid.

**Table S1** Fluid properties and

geometric parameters used in the calculation of microrobot’s motion.

| Parameter | Value | Unit |
| --- | --- | --- |
| *R* | 0.5 | mm |
| *μ* | 5 | mPa·s |
| *r* | 150 | μm |
| *θ* | 72 | ° |
| *r_w_* | 25 | μm |

**Measurement of SMA helix work energy**

Evolution of force as response to displacement is measured by Dynamic Mechanical Analysis (TA, Q800). Given that tiny structures tend to produce large errors in clamping operations, we apply helix containing several spirals to test under set procedure. The ends of the helices are clamped and then chamber was closed. Temperature ramps from 20 degrees to 65 degrees and holds for 5 minutes. After that the tensile test begins and data are recorded for further analysis.

The data columns of displacement and force of each test were selected in a table. The displacement data is divided by the helix number of the tested helix to obtain the displacement-force curve of a single helix. Integrating the displacement-force curve over a certain range, the output work energy value of helices of different helix index is obtained.


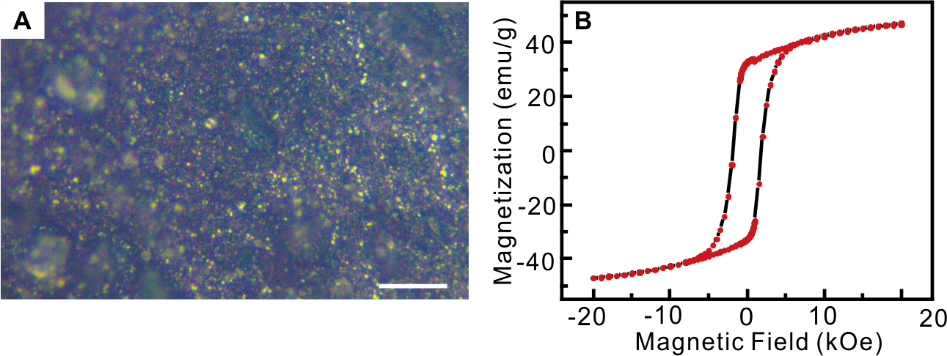


**Figure S1.** Characteristics of magnetic head**.** (A) Optical image of magnetic matrix for microrobot magnetic head. Scale bar is 50 μm. (B) Magnetization intensity as a function of applied magnetic density for PDMS embedded NdFeB particles.


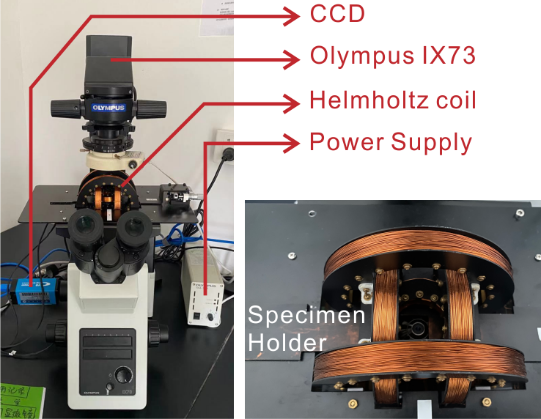


**Figure S2.** Experimental setup for rotational magnetic field generation.


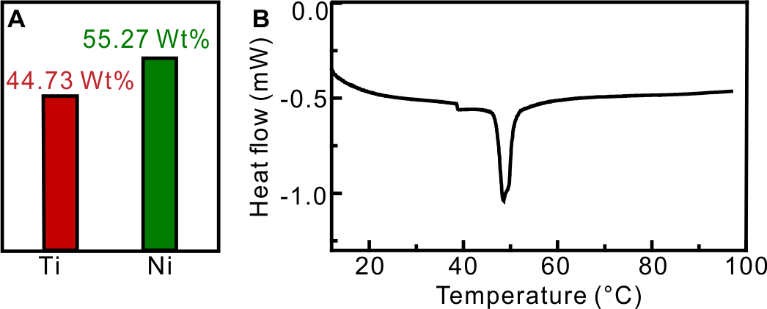


**Figure S3.** Characterization of original NiTi wire. (A) Weight ratio of Ti and Ni element. (B) DSC result.

**
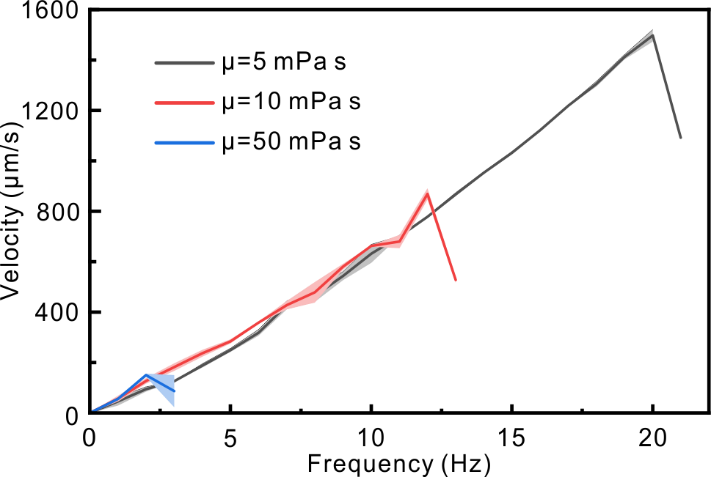
**

**Figure S4.** Translational velocity responding to applied frequency when operated in liquid with different viscosity.

**
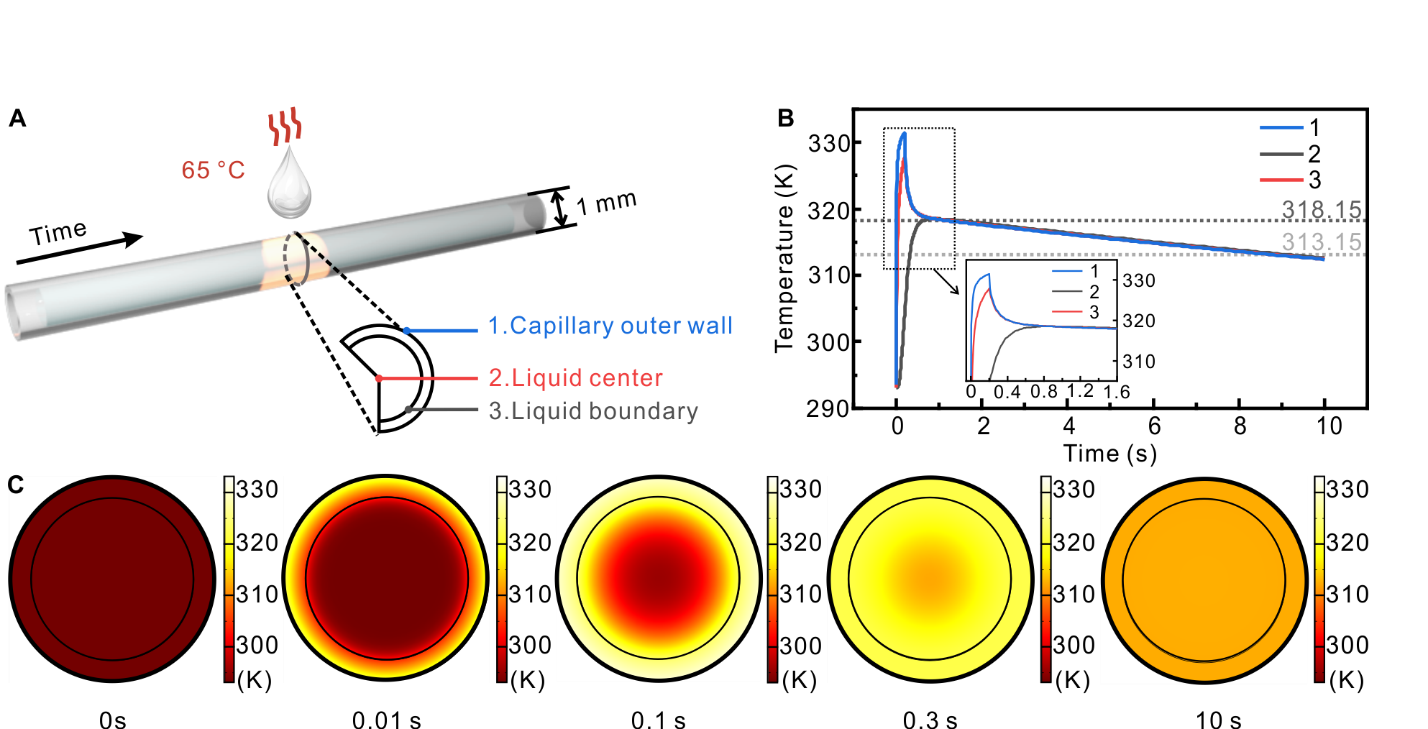
**

**Figure S5.** Analysis of heating process by adding a drop of water. (A) Schematic illustration of transformation triggering process by hot water droplet. (B) Simulation results showing temperature as a function of time at three different positions. The temperature 313.15 K and 318.15 K refers to the austenite peak temperature and phase transition complete temperature respectively. (C) Temperature field distribution of the capillary cross-section at different time.

**
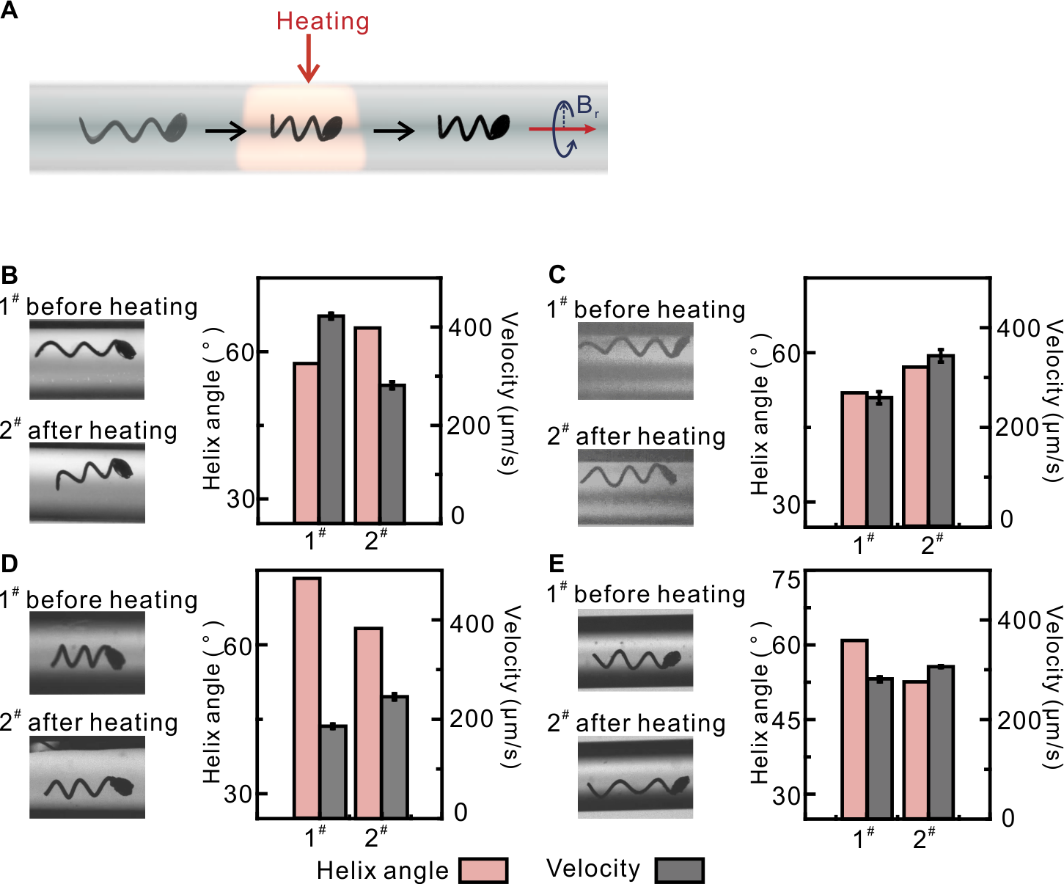
**

**Figure S6.** Real-time locomotion tuning due to shape transformation. (A) Schematic showing the real-time locomotion tuning due to shape transformation. (B-E) Velocity modulation by real-time structure transformation via heating. i-iv refers to different types of velocity modulation due to different shape transformation. Graphs present the helix angle and moving velocity of shape-memory helical microrobot (1) before and (2) after heating. In Figure S7B. the SMA helical microrobot recovers to a shorter shape resulting in velocity decreasing by around 140 μm/s. Similar recovery can also lead to increased velocity as Figure S7C. Besides, velocity change can be obtained by transforming to longer shape (Figure S7D). Apart from that, Figure S7E shows that large shape shifting can hardly change the velocity, corresponding to the two sides spanning the maximum value in Figure 3G.

**
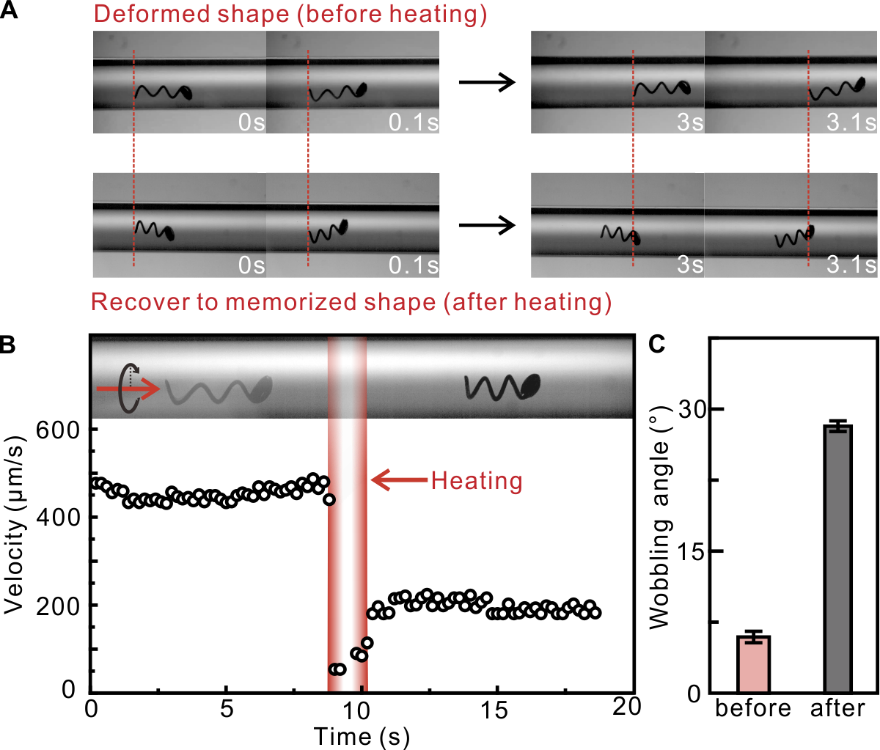
**

**Figure S7.** Gaits variation due to shape transformation of SMA helical microrobot. (A) Optical image sequences display that shape transformation leads to transformation both in velocity and wobbling angle. (B) Velocity change with respect to time. (C) Contrast of wobbling angle before and after shape transformation.


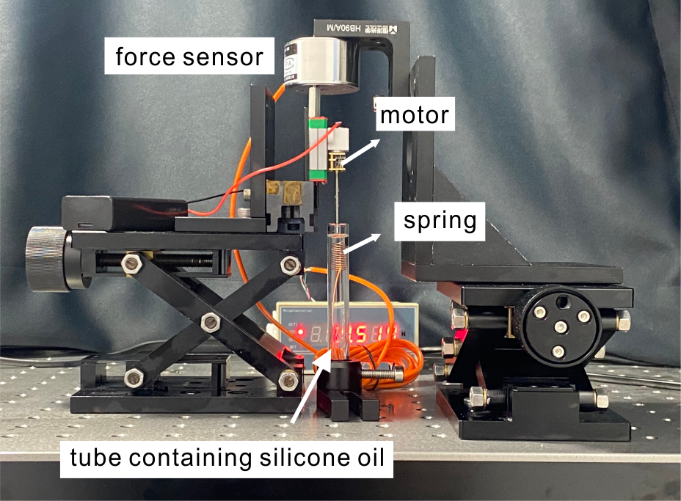


**Figure S8.** Experimental setup of various force measurement related to helix angle, including force sensor, motor, spring, and tube filled with silicone oil with dynamic viscosity of 50 mPa·s.


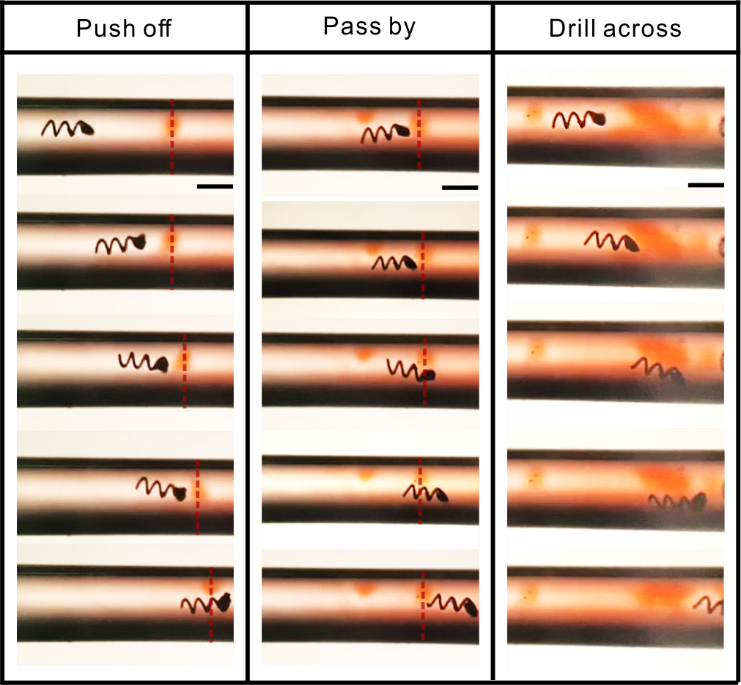


**Figure S9**. Locomotion of rigid helical microrobot in the capillary containing obstacles. The rigid microrobot reveals different strategies facing the obstacle in different size, including pushing off, passing by and drill across. Scale bar is 500 μm.


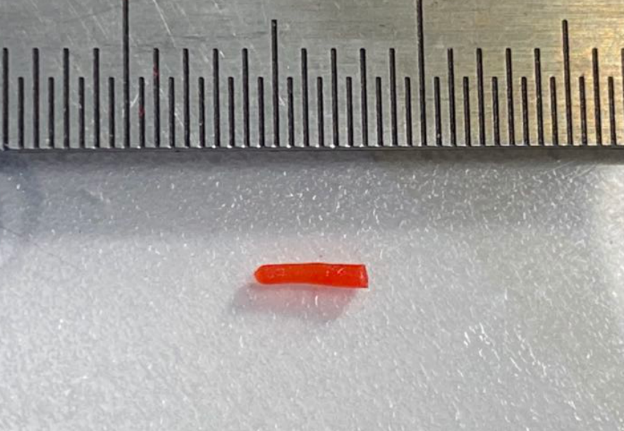


**Figure S10**. Photograph of an artificial clot.


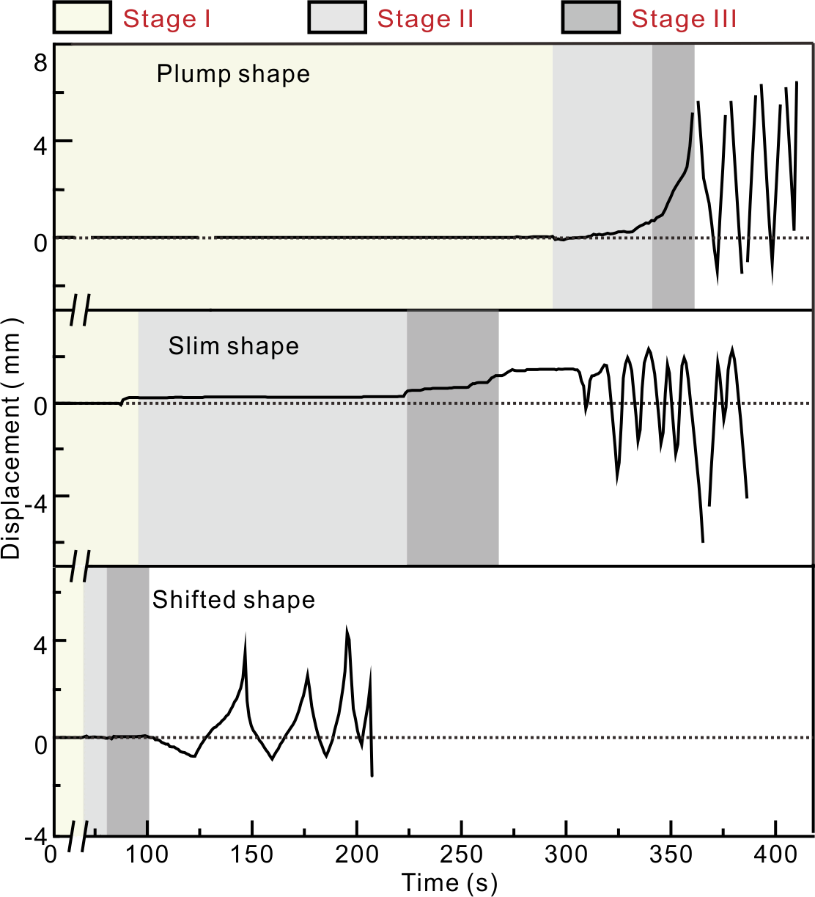


**Figure S11**. Displacement as respect to time of three unclogging strategies. Stage I to III refers to attaching the clot to drill, drilling in with one body length depth, and drilling through the clot.


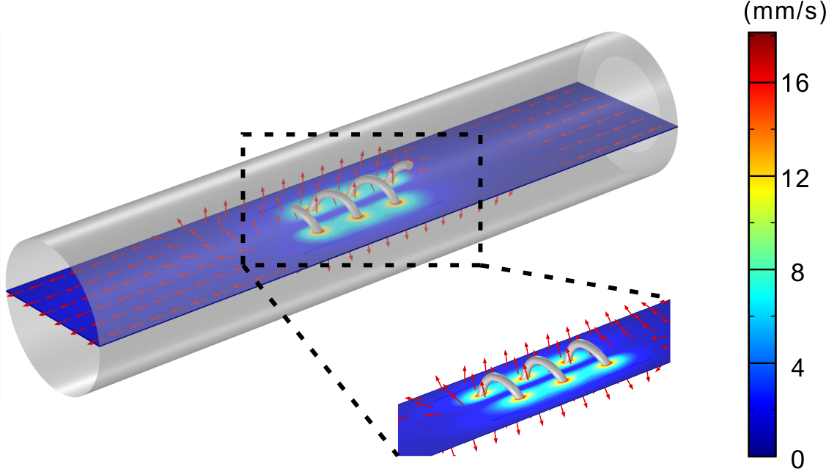


**Figure S12.** Flow field distribution when the microrobot tail rotates in low Reynolds number environment.


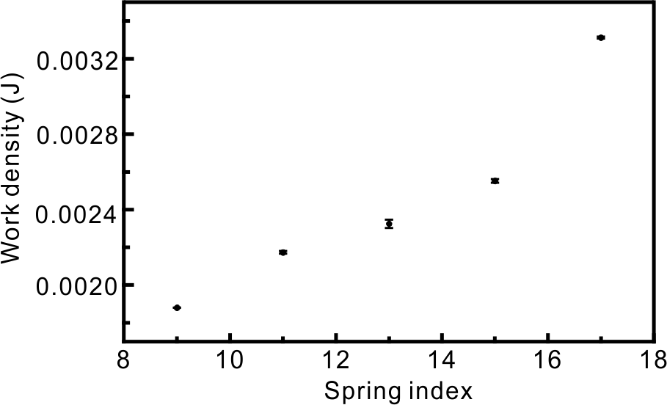


**Figure S13** Experimental result of evolution of work energy with response to spring index.


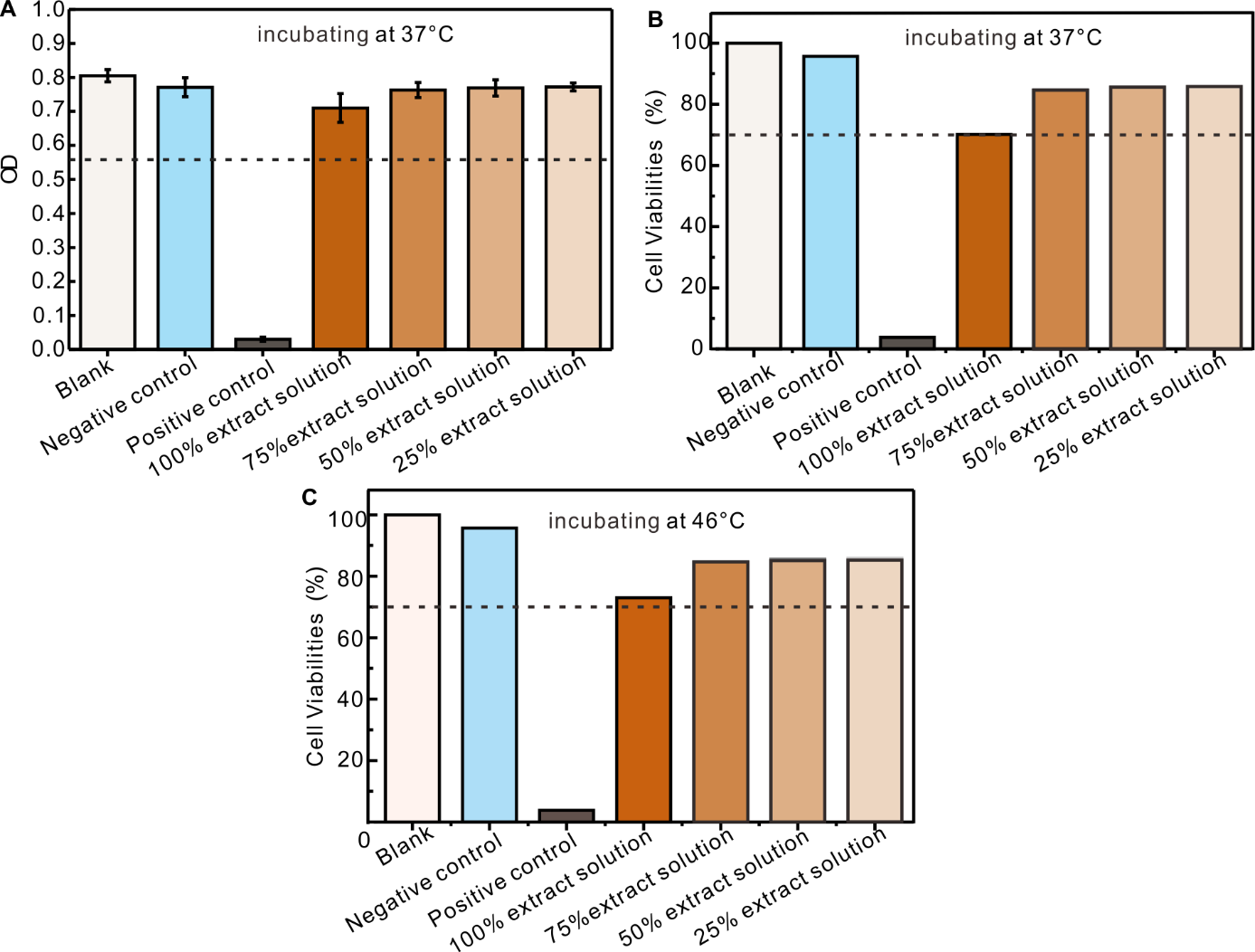


**Figure S14**. Viability of L-929 cells with SMA microrobot. (A) The extract of the test sample was cultured with vigorously growing L-929 cells (37°C, 5% CO_2_) for 24 h, then the cell morphology and cell lysis were observed, and the potential cytotoxicity of the test sample was determined by MTT method. Error bars indicate the standard deviation. (B) Cell viabilities of L-929 cells which was cultured with shape memory alloy helical microrobot for 24 h (37°C, 5% CO2). (C) Cell viabilities of L-929 cells which was cultured with shape memory alloy helical microrobot for 24 h (46°C, 5% CO_2_).


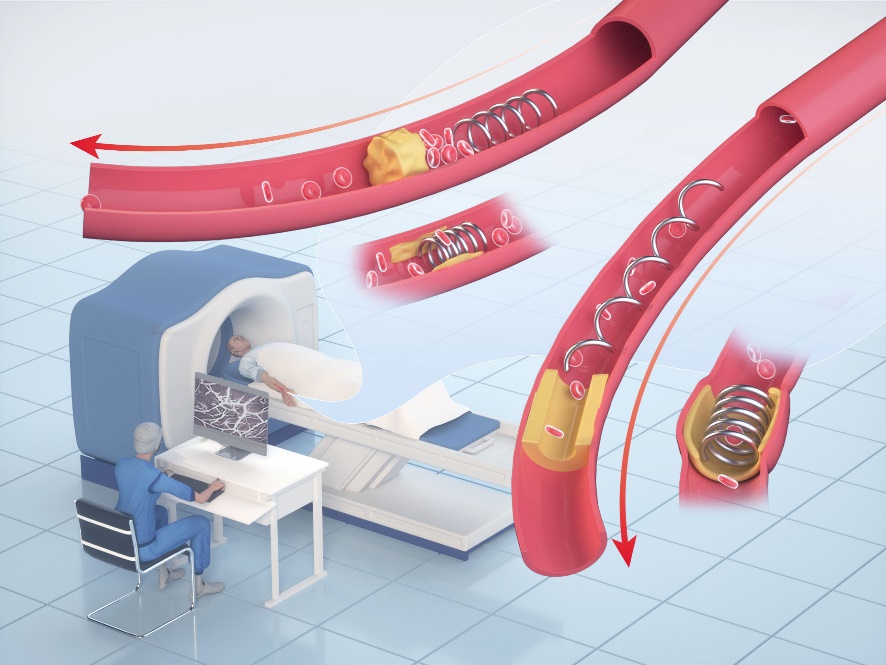


**Figure S15.** Schematic diagram of medical surgery operated by microrobot.


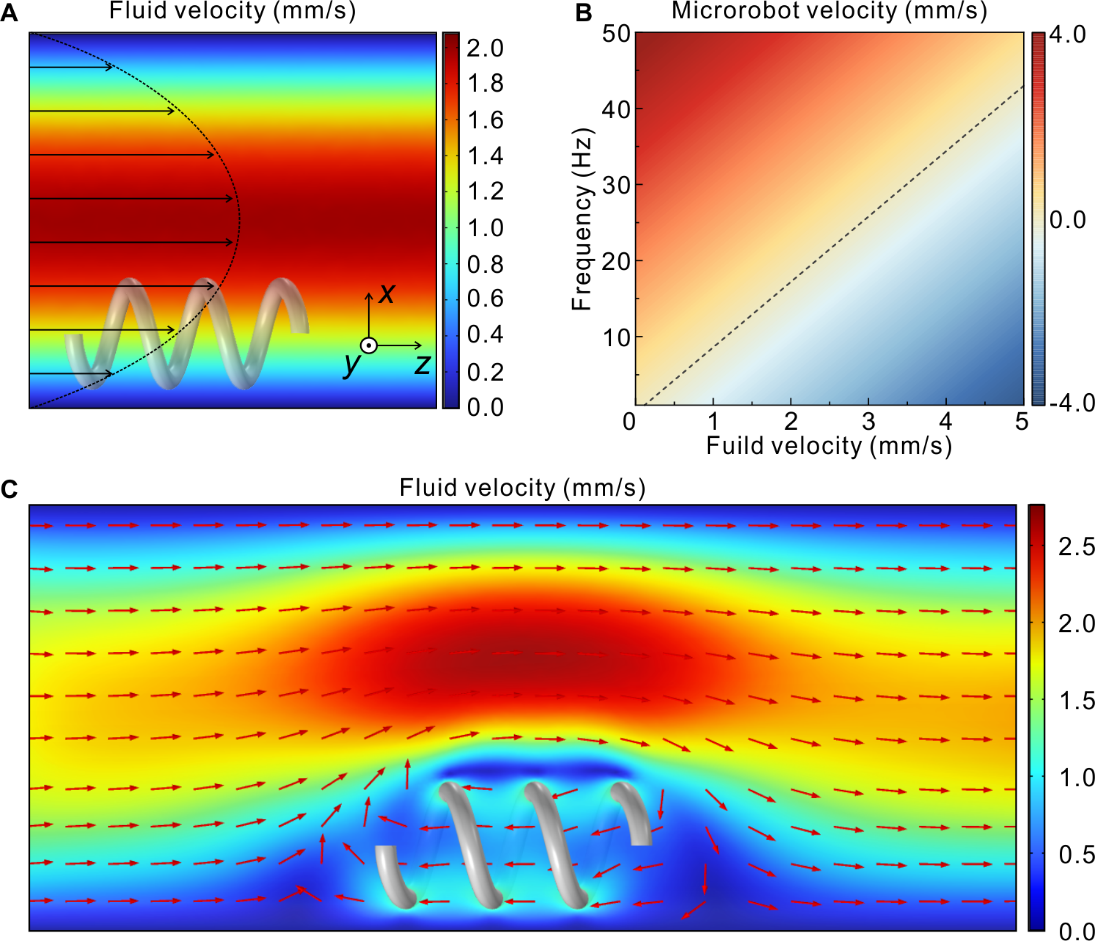


**Figure S16**. Modeling of microrobot moving against flowing liquid. (A) Fluid velocity distribution inside 1 mm-diameter channel where the average velocity of fluid is set as 1 mm/s. The image of helical microrobot depicts relative position inside the channel. (B) Calculation results of helical microrobot velocity related to the rotating frequency and fluid velocity. Dashed line refers to zero points of microrobot velocity. (C) Simulated results of fluid distribution as the helical microrobot moving against flowing liquid.

**Movie S1.** SMA helical microrobot capable of transformable ability.

**Movie S2.** The three propulsion modes with respect to frequency under same magnetic intensity.

**Movie S3.** Different velocity transformation deduced by different structure transformation.

**Movie S4.** Propulsion modes shifting caused by structure transformation.

**Movie S5.** The adaptive strategy of helical microrobot when faced with sighter clogging area.

**Movie S6.** Demonstration of enhanced unclogging effect of transformable SMA microdrill.

**Movie S7.** Demonstration of self-propelling stent.

References

41. D.J. Hartl, D.C. Lagoudas. Constitutive modeling and structural analysis considering simultaneous phase transformation and plastic yield in shape memory alloys. *Smart mater. Struct.* **18**, 104017 (2009).

42. B. Rodenborn, C.-H. Chen, H. L. Swinney, et al., Propulsion of microorganisms by a helical flagellum. *Proc Natl Acad Sci U S A* **110**, E338-347 (2013).

43. Purcell, E. M. Life at low Reynolds number. *Am J Phys* **45**, 3–11 (1977).

44. J. Lighthill. Flagellar Hydrodynamics. *SIAM Rev Soc Ind Appl Math* **18**, 161-230 (1976).
